# Genome-wide association study and haplotype analyses reveal the genetic architecture of agronomic traits and sugars in sweet sorghum

**DOI:** 10.3389/fgene.2025.1611863

**Published:** 2025-07-03

**Authors:** Abdullah Bin Umar, Ajay Prasanth Ramalingam, Bushra Sadia, Faisal Saeed Awan, Farooq Ahmad Khan, Mariam Nasir, Amy Bernardo, Paul St. Amand, Guihua Bai, P. V. Vara Prasad, Ramasamy Perumal

**Affiliations:** ^1^ Center of Agricultural Biochemistry and Biotechnology (CABB), University of Agriculture, Faisalabad, Pakistan; ^2^ Department of Agronomy, Kansas State University, Manhattan, KS, United States; ^3^ United States Department of Agriculture (USDA)-Agricultural Research Service (ARS), Hard Winter Wheat Genetics Research Unit, Manhattan, KS, United States; ^4^ Agricultural Research Center, Kansas State University, Hays, KS, United States

**Keywords:** sweet sorghum, Brix, agronomic traits, GWAS, haplotype analysis

## Abstract

**Introduction:**

Sweet sorghum is a C4 tropical grass species that has gained importance quickly as a major bioenergy crop.

**Methods:**

This genome-wide association study (GWAS) utilized a sweet sorghum panel (SSP) of 183 diverse sweet sorghum accessions genotyped by 14,819 high-quality single-nucleotide polymorphism (SNP) markers to identify novel genetic loci that are associated with major agronomic traits and sugars (Brix units, %).

**Results:**

Population stratification revealed a clear separation of the accessions based on geographical origins. The initial 50% linkage disequilibrium (LD) decay was approximately 5 kb, and the background level was approximately 80 kb, similar to that of the previously reported sorghum association panel (SAP), indicating the panel's effectiveness and reliability for GWAS. This study identified 21 significant quantitative trait nucleotides (QTNs) for the studied traits using the three (compressed)-variance component multi-locus random- SNP-effect mixed linear model (3VmrMLM), which were colocalized with previously reported quantitative trait loci (QTLs). The phenotypic variance (R2) explained by these QTNs ranged from 5.11% to 13.86%.

**Discussion:**

Additionally, haplotype analysis revealed significant phenotypic differences between haplotypes for four candidate genes, namely, *Sobic.006G128200* (a threonine-specific protein kinase gene) for days to flowering, *Sobic.001G387600* (an ethylene-insensitive gene) for Brix, *Sobic.003G069950* (a protein kinase domain gene), and *Sobic.003G214400* (an amino acid transporter gene) for fresh biomass.

## 1 Introduction

Sorghum [*Sorghum bicolor* (L.) Moench] is garnering substantial interest globally due to its versatility in providing food, feed/forage, and fuel, and it can primarily be grouped as grain, forage, and sweet sorghum (Shukla et al., 2017). Sorghum is an energy-dense C4 grass that is well-suited to the hot, semi-arid tropical environments and typically thrives well in moderately warm climates; thus, it is cultivated in many countries, including regions in Asia, Africa, Oceania, and the Americas. Grain sorghum serves as a staple food for people in the semiarid tropical regions of Africa and Asia, but it is used as livestock feed in the United States (Nasidi et al., 2019). Forage sorghum biomass is primarily used as livestock feed. Sweet sorghum has high sugar content in the stem (McCormick et al., 2018). The global production of sorghum was estimated to be 62 million metric tons (USDA, 2024). Globally, maize or corn (*Zea mays* L.) and sugarcane (*Saccharum officinarum* L.) are found to be major producers of bioethanol. Historically, sweet sorghum has been used to produce small quantities of syrup. In recent years, there has been increasing interest in its potential as a biofuel and bioenergy crop (Stamenkovic et al., 2020). In regions where sugarcane is widely cultivated, integration and addition of sweet sorghum production could potentially extend the sugar harvest period by 3–4 months (Burks et al., 2013; Rao et al., 2009; Rao et al., 2013).

Sweet sorghum is recognized as one of the most efficient sources of plant-based bioethanol produced from its sugary stalks. It is considered a promising bioenergy crop suitable for cultivation in both tropical and temperate zones and is a notable candidate for biofuel production in the United States. Compared to other bioenergy or sugar crops such as corn, sugarcane, and sugar beet (*Beta vulgaris* L.), sweet sorghum requires less water (e.g., one-third of the water needed for sugarcane and half of that for corn) and lower inputs for crop management (Almodares and Hadi, 2009). Additionally, it is relatively more tolerant to drought and salinity and produces lower greenhouse gas emissions on a life-cycle basis (Mathur et al., 2017; Kanbar et al., 2021). Its drought resilience, water-use efficiency, high temperature tolerance, and low input demands enable its cultivation on marginal annual cropland that is otherwise unsuitable for growing other food crops (Anami et al., 2015; Ramalingam et al., 2021). The juice extracted from sweet sorghum stalks can be fermented and distilled to produce bioethanol, a fuel with clean-burning properties and a high-octane rating.

Several attempts have been made to extensively market sweet sorghum globally. Sweetfuel is a consortium comprising partners from academia and industry across Europe, Brazil, India, Mexico, and South Africa with the goal to enhance yields in temperate, semi-arid, and subtropical regions through genetic improvements and better agricultural practices (Janssen et al., 2010). The potential for the swift genetic improvement in sugar yield in sweet sorghum is contingent upon a better understanding of the genetic structure of its constituent traits, Brix value (concentration of sugars), and juice volume. Bi-parental mapping populations have traditionally been employed to identify genomic regions associated with important traits in major crops (Pascual et al., 2016; Karnatam et al., 2023a). Quantitative trait loci (QTLs) influencing Brix have been identified on chromosomes 1, 2, 3, 4, 5, and 7 in various sorghum lines by linkage mapping using bi-parental populations (Lekgari, 2010; Shiringani et al., 2010; Guan et al., 2011; Felderhoff et al., 2012). However, success in bi-parental linkage mapping is often constrained by limited allelic diversity and low genomic resolution, which hampers the identification of candidate genes responsible for multiple traits (Boyles et al., 2016; Karnatam et al., 2023b). Genome-wide association studies (GWAS) can overcome those weaknesses (Sharma et al., 2023). Using GWAS, Luo et al. (2020) identified three candidate genes associated with Brix; and Burks et al. (2013) mapped QTL for sugar yield and juice volume on chromosome 6, where the *Dry midrib* (D) locus, a good predictor of sugar yield, was located.

The advent of high-throughput genotyping through next-generation sequencing (NGS) has led to the increased use of diverse association mapping panels for gene discovery. These panels are favored because they can address the significant limitations inherent in bi-parental populations. Moreover, the majority of the GWAS panels have relied heavily on germplasm derived from the sorghum conversion program, which may limit their relevance for breeding programs targeting bioenergy traits (Cuevas et al., 2017). A comprehensive understanding of allelic variation and its phenotypic effects is essential for developing superior cultivars with enhanced Brix and biomass yield. Additionally, the integration of haplotype analysis with GWAS can offer insights into the functional relevance of allelic combinations at key loci, thus further informing breeding strategies. This study addresses these gaps by assembling a diverse sweet sorghum panel having broad genetic and phenotypic variation. We employed a three (compressed) variance component multi-locus random-SNP-effect mixed-linear model (3VmrMLM), which has demonstrated superior power and accuracy in detecting quantitative trait nucleotides (QTNs), QTN-by-environment interactions (QEIs), and QTN-by-QTN interactions (QQIs) than other models (Li et al., 2022). Our hypothesis was that this diverse panel would enable the identification of novel QTNs and candidate genes associated with Brix and other agronomic traits, along with superior haplotypes through integrated haplo-pheno analysis. The specific objectives were as follows: (1) analyze allelic diversity and population structure; (2) perform GWAS for agronomic traits and Brix using 3VmrMLM; and (3) identify elite haplotypes through haplotype analysis of candidate genes.

## 2 Materials and methods

### 2.1 Materials

Diverse germplasms of 183 sweet sorghum accessions (Supplementary Table S1) collected from approximately 35 countries were acquired from the United States Department of Agriculture (USDA)—Germplasm Resource Information Network (GRIN). The study materials are hereinafter referred to as the sweet sorghum panel (SSP). All the accessions were grown in the field in the summer of 2019 and 2021 at the Directorate of Farms, University of Agriculture, Faisalabad, Pakistan (latitude 31.44′ N, longitude 73.07′ E).

### 2.2 Phenotyping for agronomic and sugar-related traits

Field morphological characterization of the SSP was carried out in two row plots for 2 years (2019 and 2021) using a randomized complete block design (RCBD) with three replications. Three uniform plants in each replication were tagged for the traits’ measurements. Agronomic traits such as days to 50% flowering (DF) were measured when 50% of the plants in each plot bloomed, days to maturity (DM) were recorded at physiological maturity before harvesting, and plant height (PH) (cm) was measured from the base of the plant to the panicle tip. Stem thickness (ST) (mm) was measured at the third internode, and the number of leaves/plant (NL) was counted at the peak vegetative stage. Fresh biomass (FB) (g) was recorded as above-ground weight per plant at harvest. Dry biomass (DB) (g) was measured after air-drying for 2 weeks after harvesting. Brix (Bx) (%) was measured after extracting juice from the stem at 75 days after planting using a handheld refractometer. Standard agronomic practices were followed throughout the cropping period. Descriptive statistics (mean, standard deviation, and coefficient of variation) and frequency distribution were performed using Minitab 21 to understand the phenotypic variability of the germplasm collection for the traits evaluated (Allen, 2019). The heritability was estimated using the metan package in R, where a mixed-linear model was fitted using the gamem() function, with genotypes as the fixed effect and replication and year as the random effects (Olivoto and Lúcio, 2020). Analysis of variance was performed using the general linear model module in R, considering variations in genotypes, year, and replication (de Mendiburu and de Mendiburu, 2017). The mean value for the traits was calculated considering both the years evaluated and was used for performing GWAS in further analysis.

### 2.3 Genotyping-by-sequencing (GBS) library preparation and sequencing

SSP was grown in controlled environmental conditions in the Department of Agronomy at Kansas State University, Manhattan, Kansas. Leaf tissues (2 cm) were collected in 96-deep-well plates at 22 days after planting, freeze-dried immediately, and ground into a fine powder for DNA isolation. Genomic DNA was isolated using the modified CTAB method (Hussain et al., 2022). DNA was quantified in a FLUOstar Omega microplate reader (BMG Labtech, Germany) using a Quant-iT™ PicoGreen dsDNA assay kit (Thermo Fisher Scientific, Waltham, MA, United States). The genomic DNA of the sorghum samples was normalized, and 200 ng per sample was used for genotyping-by-sequencing (GBS) library construction. The GBS libraries of 183 samples from the SSP were prepared using the standard protocol (Poland et al., 2012). In brief, normalized DNA was fragmented using *Pst*I and *Msp*I restriction enzymes (New England BioLabs, Ipswich, MA, United States) and ligated with barcoded adapters using a T4 DNA ligase (New England BioLabs, Ipswich, MA, United States). The barcoded DNA fragments were pooled, purified using a GenCatch PCR extraction kit (Epoch Life Science, Sugarland, TX, United States), and amplified. The PCR products were purified, and 200–300-bp amplicons were size-selected in a 2% E-gel SizeSelect II agarose gel (Thermo Fisher Scientific). The size-selected fragments were quantified in a Bio-Rad Cfx384 real-time PCR machine (Bio-Rad Laboratories, Hercules, CA, United States) using a KAPA library quantification kit (Roche Diagnostics, Indianapolis, IN, United States). Equimolar pools of the libraries were sequenced in a NextSeq 2000 sequencer using a P2 100 cycle kit (Illumina, San Diego, CA, United States) in the USDA Central Small Grain Genotyping Laboratory, Kansas State University, Manhattan, Kansas, United States.

### 2.4 SNP calling and data imputation

SNPs were called using the GBS discovery pipeline v2.0 in Trait Analysis by Association, Evolution, and Linkage (TASSEL) v5 (Bradbury et al., 2007) by aligning the sequence reads with the sorghum reference genome BTx623 (McCormick et al., 2018). Filtering using TASSEL v5 retained SNPs with ≤20% missing data, ≥0.05 minor allele frequency (MAF), and ≤0.05 maximum heterozygous proportions. Furthermore, the filtered SNPs were imputed for missing data using BEAGLE v5.0 (Browning et al., 2018). The density of the filtered SNPs in the sorghum genome was visualized using a CM plot using the R package (Yin et al., 2021).

### 2.5 Genetic structure and linkage disequilibrium measurement

By using 14,819 robust SNPs and principal component analysis (PCA), the population structure of the SSP was inferred. PCA for the SSP was aligned with the sorghum association panel (SAP) (Hu et al., 2019). A model-based maximum likelihood approach implemented by ADMIXTURE v1.23 was performed for inferring the population structure of the SSP (Alexander et al., 2009). Linkage disequilibrium (LD) decay was calculated and plotted using MaxDist 500 kb and 0.05 MAF (Zhang et al., 2019). PCA and LD decay analyses were described in detail in Ramalingam et al. (2023).

### 2.6 Genome-wide association study

Phenotypic data were integrated against the genotypic data of 14,819 SNPs to perform GWAS for identifying QTNs linked with all the traits evaluated. The 3VmrMLM model was used to perform GWAS on all nine traits using an R package (Li et al., 2022). This GWAS model simultaneously accounts for marker effects, population structure, and kinship, thereby improving the detection power and reducing false positives (Li et al., 2022). 3VmrMLM outputs results as the logarithm of the odds (LODs), which are derived based on the likelihood ratio test (LRT). Unlike traditional –log(p), which represents the p-value from standard hypothesis testing, LOD scores reflect the strength of evidence for marker trait association directly, thus providing accurate association strength within the mixed-linear model structure. In this study, QTNs with LOD ≥3 were declared significant. Significant QTNs from all traits were compared against known QTL for the related traits, and candidate genes within/closer to significant SNPs (∼50 kb) were searched using the Sorghum QTL Atlas (Mace et al., 2019) and Phytozome v13 (Goodstein et al., 2012). Haplotype analysis was performed for all the identified candidate genes, considering the SNPs retained (≥0.05 MAF and ≤0.05 maximum heterozygous proportions) in this study. All synonymous and non-synonymous SNPs were considered for haplotype analysis. Haplotypes were constructed using Haploview 4.2 (Barrett et al., 2005), employing the default block definition parameters with an LD threshold of r^2^. Haplotype frequencies were calculated and visualized in Haploview. The number of SNPs within the candidate genes ranged from two to seven. Differences in phenotypic performance across haplotypes for the candidate genes were tested using ANOVA, followed by Duncan analysis performed using Minitab (Allen, 2019). Functional annotation and pathway enrichment analysis of the candidate genes identified from GWAS were performed using ShinyGO v0.82 (http://bioinformatics.sdstate.edu/go/). The *Sorghum bicolor* genome was used as the reference, and pathway enrichment was conducted based on the Kyoto Encyclopedia of Genes and Genomes (KEGG) and Gene Ontology (GO) databases. Enrichment analysis was used to identify key biological pathways and processes that are overrepresented among the candidate genes, with statistical significance evaluated by false discovery rate (FDR)-adjusted p-values. Furthermore, candidate genes involved in significant KEGG pathways were indicated.

## 3 Results

### 3.1 Population structure and linkage disequilibrium

**FIGURE 1:**
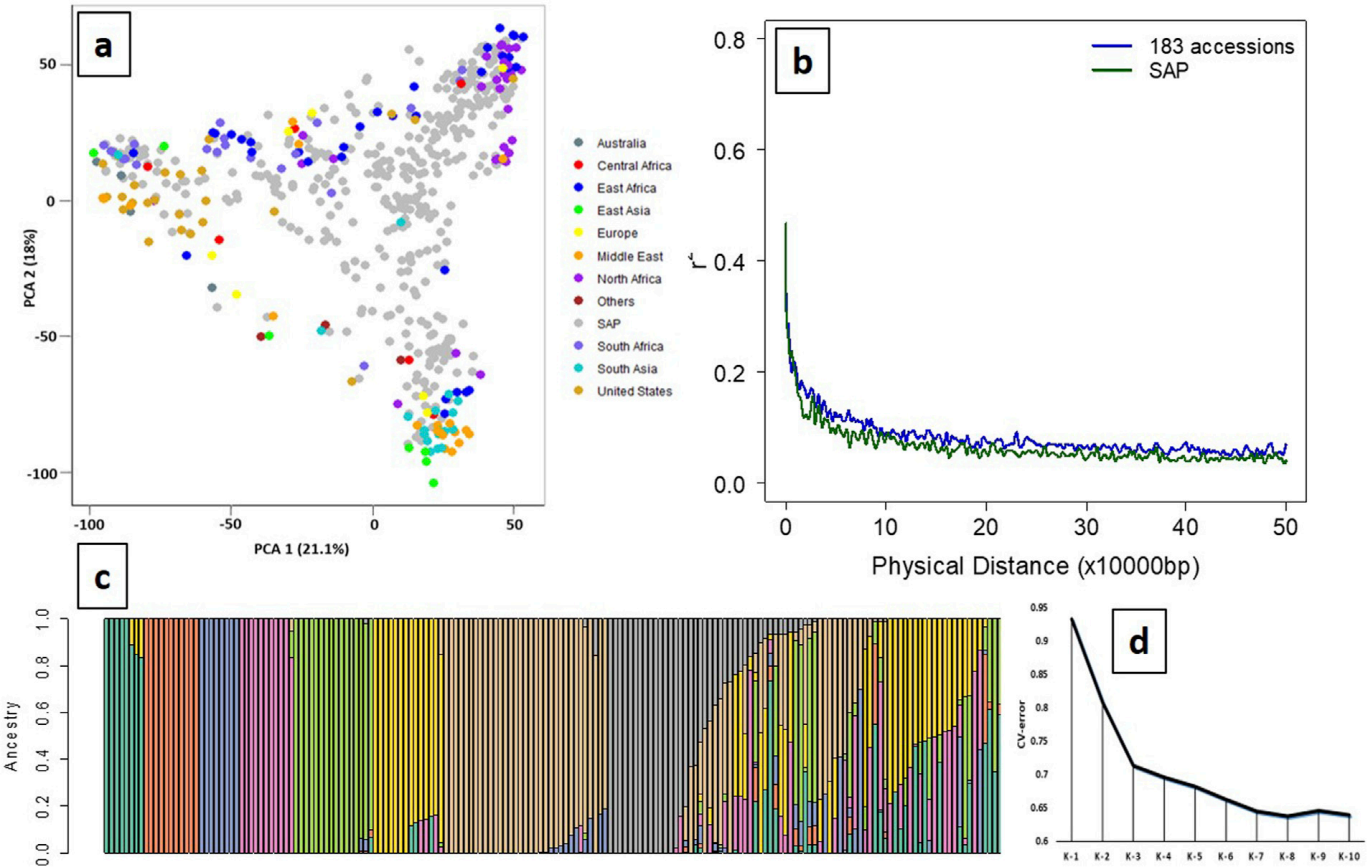
Principal component analysis (PCA), population stratification, and LD decay of the SSP (n = 183). **(a)** Principal component analysis of the SSP (n = 183) plotted on the PCA axis individually and PCA axes built with SAP (n = 401) showing wide diversity and distinct grouping based on the geographical origins. **(b)** LD decay of the SSP and SAP, indicating decay to the background level (r2 < 0.1) within ∼ 80 kb. **(c)** Population stratification using the model-based maximum likelihood approach at K = 8 showed a distinct separation between geographical origins. Green (K1), Europe; orange (K2), Middle East; blue (K3), North Africa; pink (K4), South Africa; light green (K5), South Asia; yellow (K6), United States; brown (K7), East and North Africa; gray (K8), East and South Africa. **(d)** Cross-validation (CV) error for population stratification indicated that the optimal number of subpopulations (K) for grouping the 183 sweet sorghum panel was K = 8.

A total of 14,819 high-quality SNPs exhibited wide regional diversity and were distributed in all ten chromosomes of the sorghum genome (Supplementary Figure S1). PCA of the SSP with SAP showed a cumulative variance of 39.1% using the first two PCs (21.1% and 18%), indicating a wide genetic diversity of the panel (Figure 1A). LD among the filtered SNPs (r2) used in the panel rapidly declined with increasing physical distance on the genome, with the initial 50% decay declining by ∼ 5 Kb and decay to the background level (r2 < 0.1) within ∼ 80 Kb (Figure 1B). ADMIXTURE analysis using K from 2 to 10 with tenfold cross-validation (CV) showed the minimum CV error at K = 8 (Figures 1C, D); therefore, the panel has eight subpopulation groups in this study. Distinct separation was observed among 183 accessions, with geographical origins having ancestry proportion ≥0.8 assigned to each subpopulation.

### 3.2 Phenotypic diversity

The SSP exhibited normal frequency distribution and wide phenotypic variation for the studied traits (Supplementary Figure S2). Analysis of variance was performed to assess the significance of genotype, year, replication, and genotype and environment interaction effects for all the evaluated traits (Table 1). Significant genotypic effects indicated in ANOVA support the presence of genetic variability in the germplasm, justifying the use of these evaluated traits for GWAS. Descriptive statistics revealed considerable phenotypic variation across the SSP (Supplementary Table S2). All the traits showed moderate-to-high broad-sense heritability (0.45–0.94), with the lowest for PH and the highest for DF (Supplementary Table S2). The moderate-to-high heritability facilitated the identification of potential QTNs for these traits through GWAS.

**TABLE 1:** Analysis of variance indicating significant genetic variability in the germplasms for the traits evaluated.

Source	PH	NL	DF	DM	FB	DB	BX	ST
Genotypes	6,323*	14.93*	1,430.8*	323*	40,289*	2,878*	39.3*	83.62*
Environment	273	2.55	421.1*	5,430*	178,404	19,096	321.3	141.38
Replication	37	1.67	2.1	2.5	42,473	29,305	18.5	0.63
G X E	222	7.39	18.2*	20*	11,092	3,966	4.2	2.09
Error	35	1.33	0.3	0.1	11,953	3,978	0.6	0.72

*indicate significance at p ≤ 0.01.

PH, plant height; NL, number of leaves/plant; DF, days to flowering; FB, fresh biomass; DB, dry biomass; Bx, Brix content; ST, stem thickness.

### 3.3 Genome-wide association study

**FIGURE 2:**
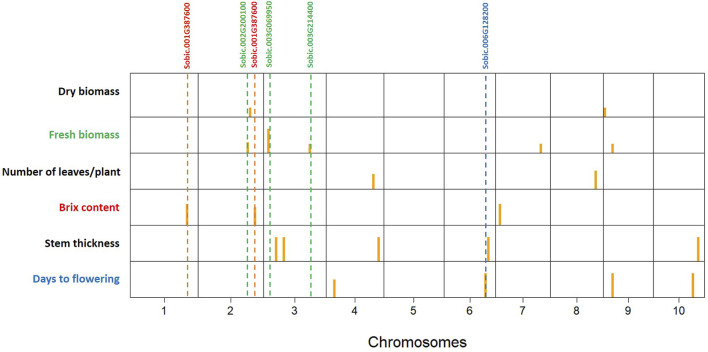
Significant quantitative trait nucleotides (QTNs) mapped by genome-wide association studies (GWAS) for all the traits evaluated on the sweet sorghum panel (SSP) using the 3VmrMLM model. Yellow bars are QTN positions in the chromosomes (1–10). The putative candidate genes are listed at the top, and the dashed lines show the relative positions of QTNs with the candidate genes.

The 3VmrMLM model detected 21 QTNs associated with different traits that we evaluated, and they were localized within, or in proximity to, one of the 19 candidate genes (Tables 2, 3; Supplementary Table S3; Supplementary Figure S3). The number of QTNs detected for each trait ranged from two to five, with phenotypic variance (R^2^—R-squared) ranging from 5.11 to 13.86. For four significant QTNs detected for DF, S06_49330184 was in a putative candidate gene; *Sobic.006G128200* had R^2^ = 6.45 (Figure 2); whereas a candidate gene for DF was not found in proximity to S10_47190193. Among the five significant stem thickness-related QTNs, S10_53386896 was within *Sobic.010G192200* with R^2^ = 6.16. Two of three significant Bx QTNs were localized within candidate genes *Sobic.001G387600* and *Sobic.002G295800*.

**TABLE 2:** Number of QTNs detected in the traits evaluated in this study.

Trait	Number of QTNs
Days to flowering	4
Stem thickness	5
Brix content	3
Number of leaves/plant	2
Fresh biomass	5
Dry biomass	2

**TABLE 3:** Key candidate genes identified in proximity to the significant quantitative trait nucleotides (QTNs).

Trait	QTN	Chromosome	Position	P-value	R2	Candidate gene	Annotation	Base pairs away
Days to flowering	S04_9611322	4	9611322	4.61E-06	9.33	*Sobic.004G102700*	BES1/BZR1 homolog protein, putative, expressed	20,873
	S06_49330184	6	49330184	0.000314	6.45	*Sobic.006G128200*	Threonine-specific protein kinase	0
	S09_10767169	9	10767169	4.79E-05	7.50	*Sobic.009G080000*	SQUAMOSA PROMOTER-BINDING-LIKE PROTEIN 14-RELATED	23,015
	S10_47190193	10	47190193	1.54E-05	6.10	*NA*	NA	NA
Stem thickness	S03_14723140	3	14723140	2.88E-10	5.11	*Sobic.003G144400*	Homeodomain-like	21,212
	S03_23932008	3	23932008	2.25E-11	8.75	*Sobic.003G165800*	UDP-N-acetylglucosamine--N-acetylmuramyl-pyrophosphoryl-undecaprenol N-acetylglucosamine transferase, putative, expressed	261,094
	S04_62493744	4	62493744	1.72E-06	5.48	*Sobic.004G283201*	AP2 domain	5,707
	S06_52891675	6	52891675	4.21E-05	6.34	*Sobic.006G173000*	Transporter, major facilitator family	3,203
	S10_53386896	10	53386896	0.000131	6.16	*Sobic.010G192200*	Two-component response regulator ARR-B family	0
Brix content	S01_67476108	1	67476108	2.49E-05	8.92	*Sobic.001G387600*	Ethylene-insensitive 3	0
	S02_67296984	2	67296984	5.35E-13	7.97	*Sobic.002G295800*	Protein kinase domain	0
	S07_5383507	7	5383507	0.000409	6.72	*Sobic.007G053100*	Amino acid permease family protein	9,810
Number of leaves/plant	S04_56429277	4	56429277	4.85E-06	8.06	*Sobic.004G214500*	DNA repair protein RAD18	1,036
	S08_53828669	8	53828669	1.71E-06	6.03	*NA*	NA	NA
Fresh biomass	S02_58995294	2	58995294	1.14E-05	5.53	*Sobic.002G200100*	Zinc finger, C2HC5-type	0
	S03_5989832	3	5989832	5.99E-10	7.05	*Sobic.003G069950*	Protein kinase domain	0
	S03_54843288	3	54843288	2.09E-07	10.19	*Sobic.003G214400*	Amino acid transporter	0
	S07_53586588	7	53586588	1.32E-05	7.04	*Sobic.007G125400*	Chitinase	51,877
	S09_11063229	9	11063229	3.59E-13	8.97	*Sobic.009G080400*	GLUCOSYL/GLUCURONOSYL TRANSFERASES	2,162
Dry biomass	S02_61546596	2	61546596	2.57E-17	7.91	*Sobic.002G224100*	Phosphate carrier protein, mitochondrial precursor	6,023
	S09_1695932	9	1695932	6.27E-08	13.86	*Sobic.009G018450*	Threonine-specific protein kinase	246

Between two significant QTNs for NL, S04_56429277 was in proximity to *Sobic.004G214500*, a candidate gene related to leaf number. Among five significant FB QTNs, three were colocalized with the putative genes related to biomass, and S03_54843288, with the highest R^2,^ was localized within the gene *Sobic.003G214400*. For DB, two significant QTNs were located in proximity to candidate genes related to biomass, and one of them, S09_1695932, with a higher R^2^ value (13.86), was in close proximity to *Sobic.009G018450*.

### 3.4 Candidate gene haplotype analysis

**FIGURE 3:**
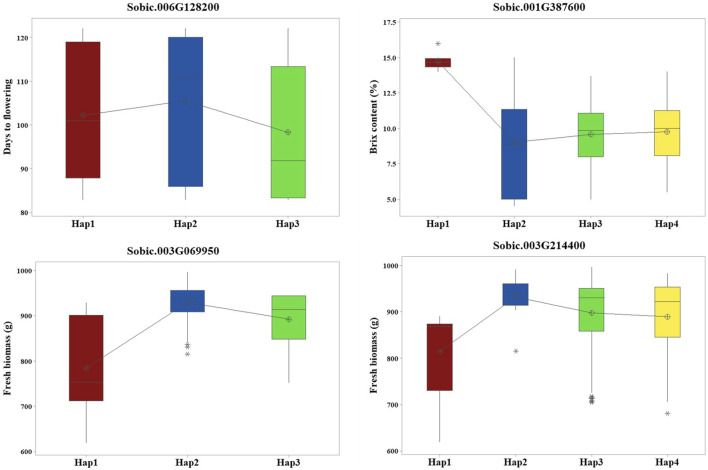
Haplotype analysis revealed four candidate genes with significant phenotypic differences among their respective haplotypes.

Haplotype analysis performed on 19 candidate genes with their associated SNPs found that four candidate genes showed significant phenotypic differences (p < 0.05) between the haplotypes (Figure 3). *Sobic.006G128200* had two SNPs that formed three haplotypes, which showed clear differences in flowering time. *Sobic.001G387600* had seven SNPs forming four haplotypes linked to differences in Brix. For FB, *Sobic.003G069950* had four SNPs making three haplotypes, and *Sobic.003G214400* had three SNPs forming four haplotypes, all showing differences in trait values (Supplementary Tables S4, 5).

### 3.5 Gene enrichment and pathway analysis of candidate genes

Functional enrichment analysis of the candidate genes identified from GWAS revealed six significantly enriched pathways (Table 4). Enriched pathways included zeatin biosynthesis (FDR = 1.2E-04), MAPK signaling pathway-plant (FDR = 7.5E-10), and plant–pathogen interaction (FDR = 8.2E-09). Additionally, pathways involved in amino sugar and nucleotide sugar metabolism, plant hormone signal transduction, and biosynthesis of nucleotide sugars were significantly overrepresented. These pathways were characterized by high fold enrichment values ranging from 2.5 to 5.4, indicating a strong association of the candidate genes with metabolic and signaling processes, potentially influencing agronomic traits and Brix content.

**TABLE 4:** KEGG pathways associated with the candidate genes identified for agronomic traits and Brix content.

Enrichment FDR	Fold enrichment	Pathway	Candidate genes involved in the pathway
0.000122135	5.388943489	Path:sbi00908, zeatin biosynthesis	*Sobic.002G200100, Sobic.002G224100, and Sobic.009G018450*
7.5012E-10	4.704633205	Path:sbi04016, MAPK signaling pathway-plant	*Sobic.007G125400 and Sobic.002G224100*
8.22332E-09	3.42711492	Path:sbi04626, plant–pathogen interaction	*Sobic.004G102700, Sobic.009G080000, Sobic.006G128200, and Sobic.004G214500*
1.06618E-05	3.010965251	Path:sbi00520, amino sugar and nucleotide sugar metabolism	*Sobic.009G080400, Sobic.002G295800, and Sobic.007G053100*
1.06618E-05	2.599928876	Path:sbi04075, plant hormone signal transduction	*Sobic.009G018450, Sobic.004G214500, Sobic.006G128200, and Sobic.002G295800*
0.023790134	2.49832246	Path:sbi01250, biosynthesis of nucleotide sugars	*Sobic.001G387600, Sobic.002G295800, and Sobic.007G053100*

## 4 Discussion

Compared to other grasses, sorghum is notable for its adaptability, versatility, superior water-use efficiency (Prasad et al., 2007; Prasad et al., 2021), and temperature tolerance (Prasad et al., 2017). The crop is grown annually, exhibiting rapid growth and excellent climate adaptability (Reddy et al., 2007). It has potential for use in biofuel production due to its high content of fermentable sugars and biomass (Stamenkovic et al., 2020). Brix value, which measures the sugar concentration (%) in the plant’s juice, is a critical trait for sweet sorghum, particularly for bioethanol production. High Brix values indicate a higher concentration of fermentable sugars and higher ethanol yields (Wu et al., 2010). Plant height, directly related to biomass yield, is another critical trait in sweet sorghum (Prasanth et al., 2021). The selection of superior sweet sorghum accessions based on key traits such as Brix value and plant height is crucial for improving biofuel yield and overall crop performance (Mathur et al., 2017). We found a significant positive correlation between plant height and FB (data not presented), which is in agreement with the findings by Yamazaki et al. (2020). For example, five potential accessions, namely, PI 152630, PI 167352, PI 152880, PI 157033, and PI 170802, showed the highest Brix (13%–16%) with the average plant height of 266 cm (medium tall), and the other five accessions (PI 641807, PI 152683, PI 566819, PI 152961, and PI 180348) with the low Brix (5%–8%) only had the average plant height of 181 cm (medium) (Supplementary Table S1). Accessions from these two groups of extreme genotypes can be used to develop bi-parental mapping populations by crossing accessions between the groups to validate the GWAS-based candidate genes identified in this study.

The high level of genomic similarity between sweet and grain sorghum has been shown in previous studies. Cooper et al. (2019) identified this genomic similarity using the reference genome BTx623, an early-maturing and short-grain sorghum line, against “Rio,” a sweet sorghum line. Rio is genotypically more similar to BTx623 than to some other sweet sorghum accessions, but they show significant phenotypic differences. In this study, a total of 14,819 filtered high-quality SNPs were used for PCA, and the results indicated a well-scattered distribution of the sweet sorghum accessions along with the SAP (Figure 1A). It clearly indicated that the selected 183 sweet sorghum accessions used in this study covered a greater genetic diversity and, hence, can be used as the SSP for future genomic studies.

Sorghum has been categorized into five major races: bicolor, caudatum, durra, guinea, and kafir, and the categories are primarily based on panicle and grain characteristics and their regions of origin in Africa and India (Harlan and deWet, 1972). Since sweet sorghum has not been selected for panicle or grain traits, and its origins provide limited insights, its relationship to the major sorghum races defined based on the traditional classification remains inconsistent. This study utilized a sweet sorghum collection of diverse origins (race details unknown) and revealed population stratification into eight distinct groups using a model-based maximum likelihood approach (Figure 1C). These groups corresponded closely to geographical origins, reflecting the natural genetic structure of the germplasm. For example, K1 (green) predominantly included accessions from Europe, K2 (orange) from the Middle East, K3 mainly from North Africa, K4 primarily from South Africa, K5 largely from South Asia, K6 from the USA and Middle East, K7 from North and East Africa, and K8 mainly from South and East Africa. Furthermore, the LD decay pattern in our study population resembled that of the SAP, suggesting comparable genome-wide linkage patterns. While the 14,819 filtered SNPs do not directly confirm complete genomic coverage (Supplementary Figure S1), our study supports the notion that the filtered SNPs are robust and appropriate for GWAS analysis. Collectively, these observations support the reliability of the SNP dataset and the potential utility for sweet sorghum breeding and genomic applications.

The GWAS captures the additive genetic variance, thereby elucidating the genetic basis of complex traits (Caballero et al., 2015). Consequently, heritability serves as a crucial parameter in predicting the power of gene mapping in GWAS to some extent. Heritability (H^2^) reflects the proportion of phenotypic variation due to genetic factors and not just the total variation observed. While plant height and number of leaves show high phenotypic variability, much of this variation is likely due to environmental effects, leading to lower H^2^. In contrast, biomass traits have lower total variability but a higher proportion of genetic variance, resulting in higher heritability in this study. The GWAS on 183 sweet sorghum accessions was performed to identify the genetic loci controlling Brix and other agronomic traits. Several statistical models can be used for GWAS, but only a few have been demonstrated to have high accuracy and power in mapping loci (Wang and Zhang, 2021). The traits such as plant height and days to maturity showed bimodal distributions, and significant QTNs were not detected for these traits, which may be due to limited genetic background effects (Han et al., 2021) (Supplementary Table S3; Figure 2). 3VmrMLM provides a multilocus framework that improves detection power while reducing false positives, making it more suitable for dissecting complex traits in our SSP. The 3VmrMLM model has proven to be effective in detecting all types of loci by encompassing QEIs and QQIs (Li et al., 2022; Ramalingam et al., 2023; Mohanavel et al., 2025). This model can estimate their effects almost without bias, maintaining high accuracy and power with a low false-positive rate. In this model, QTNs were represented by pink dots (Supplementary Figure S3), and they have strong and independent effects on the traits studied. While some SNPs may appear statistically significant on their own, they are not retained as significant QTNs in 3VmrMLM as they did not provide additional explanatory power when considered alongside QTNs. This multi-locus approach ensured that only the most robust associations were selected in this study. In this investigation, the majority of the QTNs were found to co-localize with previously reported QTLs, thereby validating some of these loci and candidate genes using a diverse sweet sorghum germplasm collection and advanced GWAS models, including 3VmrMLM. Additionally, the integration of haplotype analysis provided further insights into allelic variation at key candidate genes and its impact on agronomic traits, such as biomass and Brix. This approach enhances our understanding of the genetic architecture underlying these traits and offers valuable information for sorghum breeding programs aimed at improving the yield and sugar content.

The flanking sequences of the identified QTNs were searched against the sorghum QTL atlas database, which identified 19 candidate genes associated with the different traits evaluated. The functional relevance of these candidate genes was further explored through haplotype analysis. Days to flowering was associated with S06_49330184. S06_49330184 is a significant QTN within *Sobic.006G128200*, which is a threonine-specific protein kinase. This putative candidate gene showed phenotypic difference in flowering between different haplotypes formed. Similarly, the significant QTN S10_53386896, which is associated with stem thickness, was localized within *Sobic.010G192200,* encoding a two-component response regulator of the ARR-B family. These regulators are key components of cytokinin signaling, which influences shoot development and vascular differentiation in plants (Xie et al., 2018). The proximity of this gene to the QTN, along with its functional annotation, highlights its potential role in modulating stem thickness, which is a critical trait for biomass and structural integrity.

Sugar yield-related traits, such as juice volume and Brix, are affected by genotypic, environmental, and genotype-by-environment effects and are quantitatively inherited (Shiringani et al., 2010). GWAS for Brix identified three candidate genes, including S01_67476108, located approximately 1.2 Mb from *SbSUT1*, a well-characterized sugar transporter in sorghum (Milne et al., 2017). In addition to the proximity of the gene with a known function, this QTN is localized within *Sobic.001G387600* encoding ethylene-insensitive 3. Different haplotypes of this gene showed significant differences in Brix, with Hap1 (AGGATAA) identified as the superior haplotype associated with a higher mean for Brix (Supplementary Tables S4, 5). Other candidate genes detected for Brix include *Sobic.002G295800* and *Sobic.007G053100*, and they are localized within the reported QTLs, *QSUGY2.2* and *QBRIX7.1*, respectively (Shiringani et al., 2010).

QTNs were detected for the number of leaves/plants, FB, and DB, among which S03_5989832 and S03_54843288 were localized within the candidate genes *Sobic.003G069950* and *Sobic.003G214400*, respectively. *Sobic.003G069950* encodes a protein kinase domain-containing protein, which is homologous to kinases in rice known to regulate biomass accumulation (Liu et al., 2015). *Sobic.003G214400* encodes an amino acid transporter implicated in plant growth regulation in rice (Ji et al., 2020) and colocalizes with *QFBMS3.4*, a previously reported QTL for biomass in sorghum (Shiringani and Friedt, 2011). Furthermore, haplotype analysis of these genes indicated significant phenotypic differences in FB. *Sobic.003G069950* exhibited three haplotypes with significant differences in FB, among which Hap2 (AATT) was identified as the superior haplotype, showing the highest mean biomass of 929.5 g. Similarly, in *Sobic.003G214400*, Hap2 with the AAG allelic combination showed greater mean biomass, indicating its superiority (Supplementary Tables S4, 5). These identified superior haplotypes hold potential for use in sorghum breeding programs upon further validation.

Pathway enrichment analysis revealed that amino sugar and nucleotide sugar metabolism were significantly enriched among the candidate genes identified from GWAS (Supplementary Figure S4). This pathway plays a crucial role in the biosynthesis and interconversion of nucleotide sugars, which are essential precursors for polysaccharides and glycoproteins (Ginsburg, 2009). Specifically, genes involved in the conversion of glucose-6-phosphate and fructose-6-phosphate into UDP-glucose and UDP-galactose may influence the accumulation of soluble sugars within the stem, which is reflected in Brix measurements. Enriched pathways such as amino sugar and nucleotide sugar metabolism not only indicate a direct biochemical link to the Brix content but also suggest a potential regulatory role in sugar partitioning and accumulation (Ginsburg, 2009).

Although the candidate genes showed phenotypic differences between the haplotypes via haplotype analysis, their importance and value need to be confirmed through linkage mapping and differential gene expression. In this study, other important sugar-related traits, including juice, sugar, and ethanol yield; concentrations of sucrose, glucose, and fructose; and the total soluble sugar content suggested by Ekefre et al. (2017) were not included, as phenotyping of these traits with a large number of germplasms is cumbersome and challenging. These kinds of limitations can be overcome by a follow-up detailed evaluation in future studies of these traits by using a narrow set of selected SSP germplasms. In summary, this study provides an SSP for mapping the complex agronomic traits of economic importance, which are under polygenic inheritance. Furthermore, the significant QTNs and candidate genes identified in this study can be utilized for trait introgression or stacking through marker-assisted backcrossing into elite sorghum parental lines after validation. Creating bi-parental mapping populations by crossing elite sorghum lines with accessions of extreme performance from the panel possessing agronomic and Brix alleles will facilitate the mapping of genomic regions for validation in future studies. Other validation studies to consider in future studies include RNA sequencing, qRT-PCR validation, and Kompetitive Allele-Specific PCR (KASP) marker development.

## Data Availability

The genomic data of the subset generated in this study have been deposited into the NCBI database under accession code PRJNA1256919.
