# Supplementary material for: Genome-wide association study and haplotype analyses reveal the genetic architecture of agronomic traits and sugars in sweet sorghum
Source: Front Genet. 2025 Jul 3;16:1611863. doi: 10.3389/fgene.2025.1611863 (PMC12267045; doi:10.3389/fgene.2025.1611863)
Supplement: Supplementary file 1 [file DataSheet1.docx]

Supplementary Materials

# Supplementary Figures


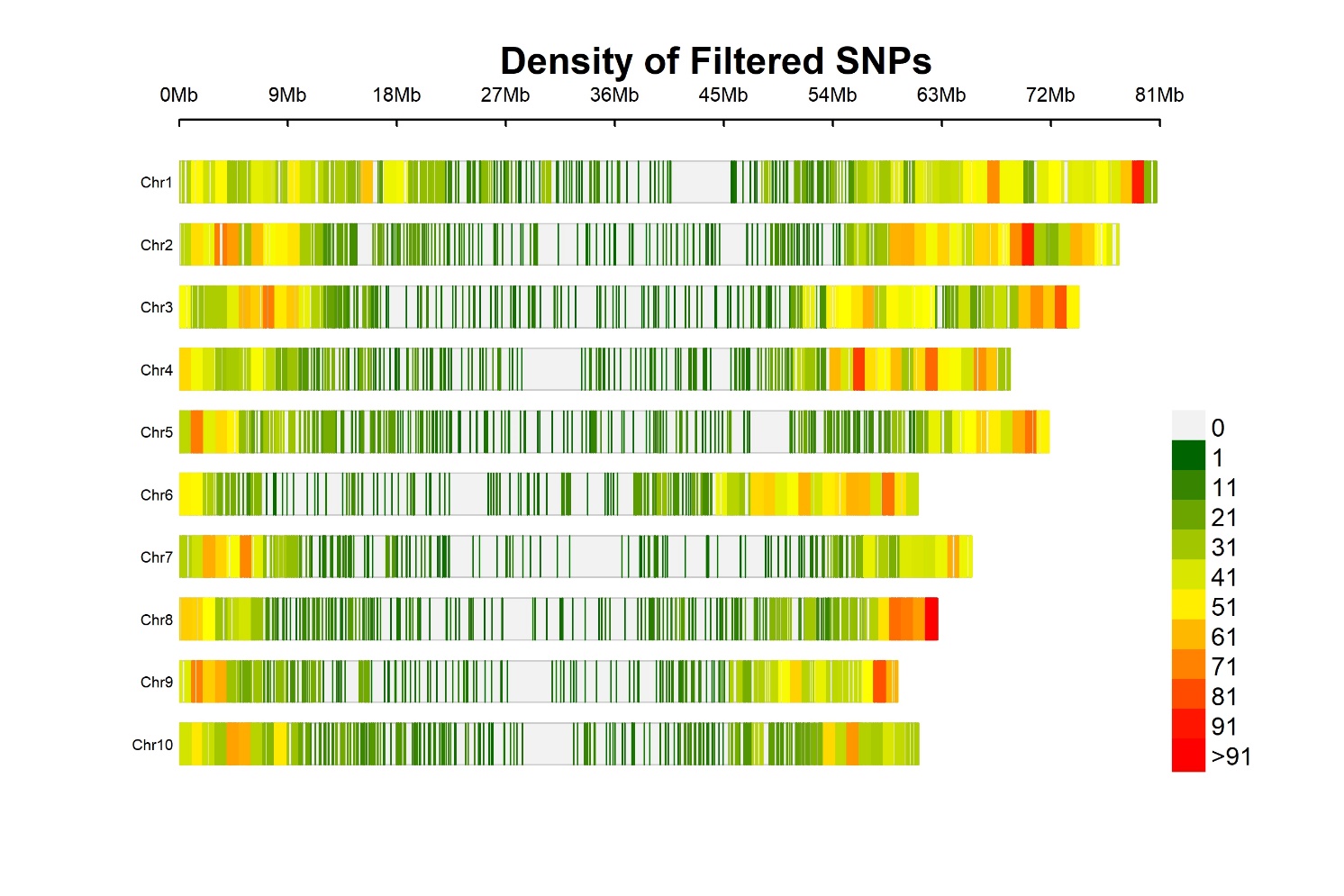


**Supplementary Figure 1.** Chromosome wise distribution of filtered robust 14819 SNPs used in this study.


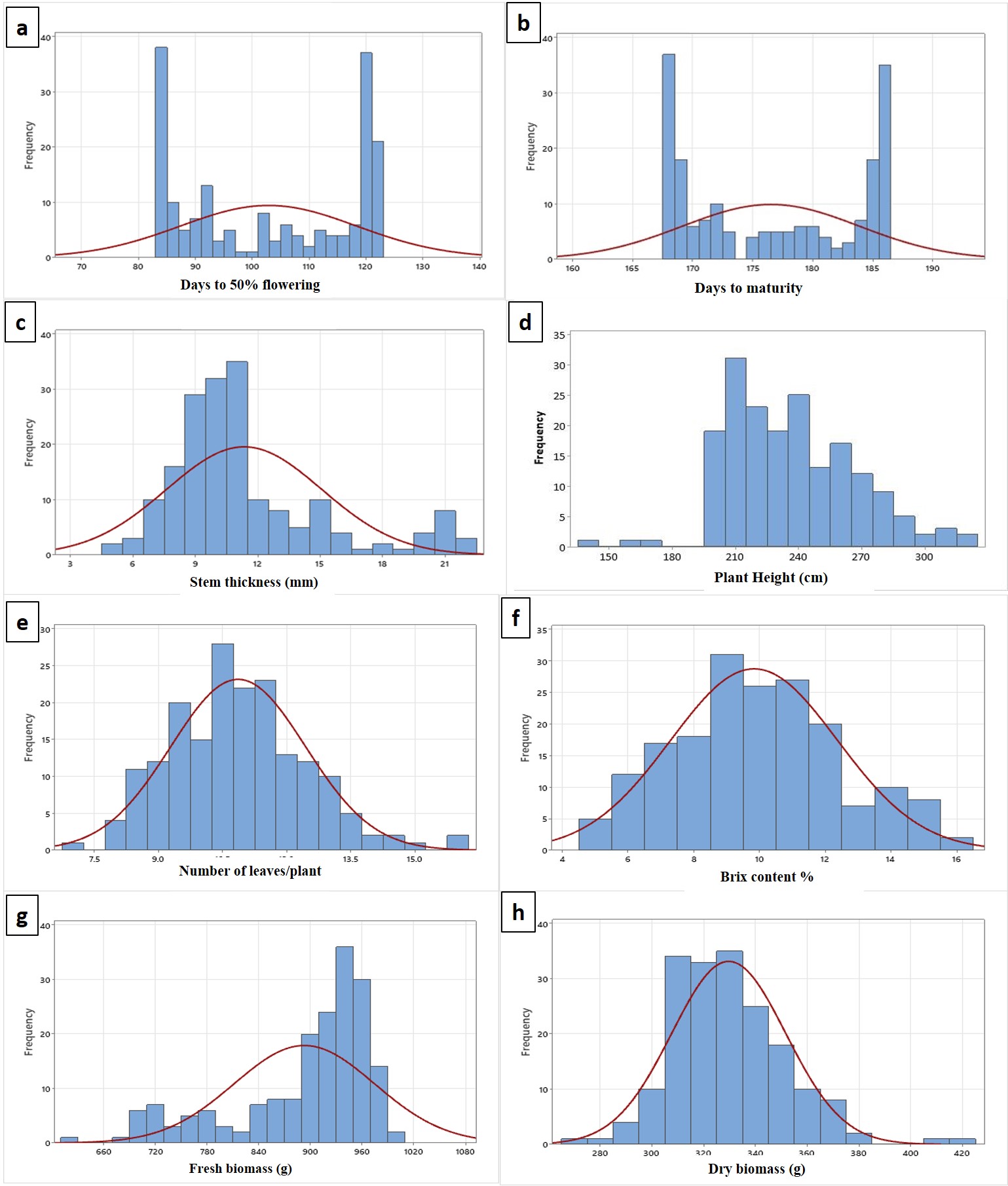


**Supplementary Figure 2.** Frequency distribution of the sweet sorghum panel to show continuous variation for all eight traits. a. Days to flowering, b. days to maturity, c. stem thickness, d. plant height, e. number of leaves/plant, f. Brix content, g. fresh biomass and h. dry biomass.

**
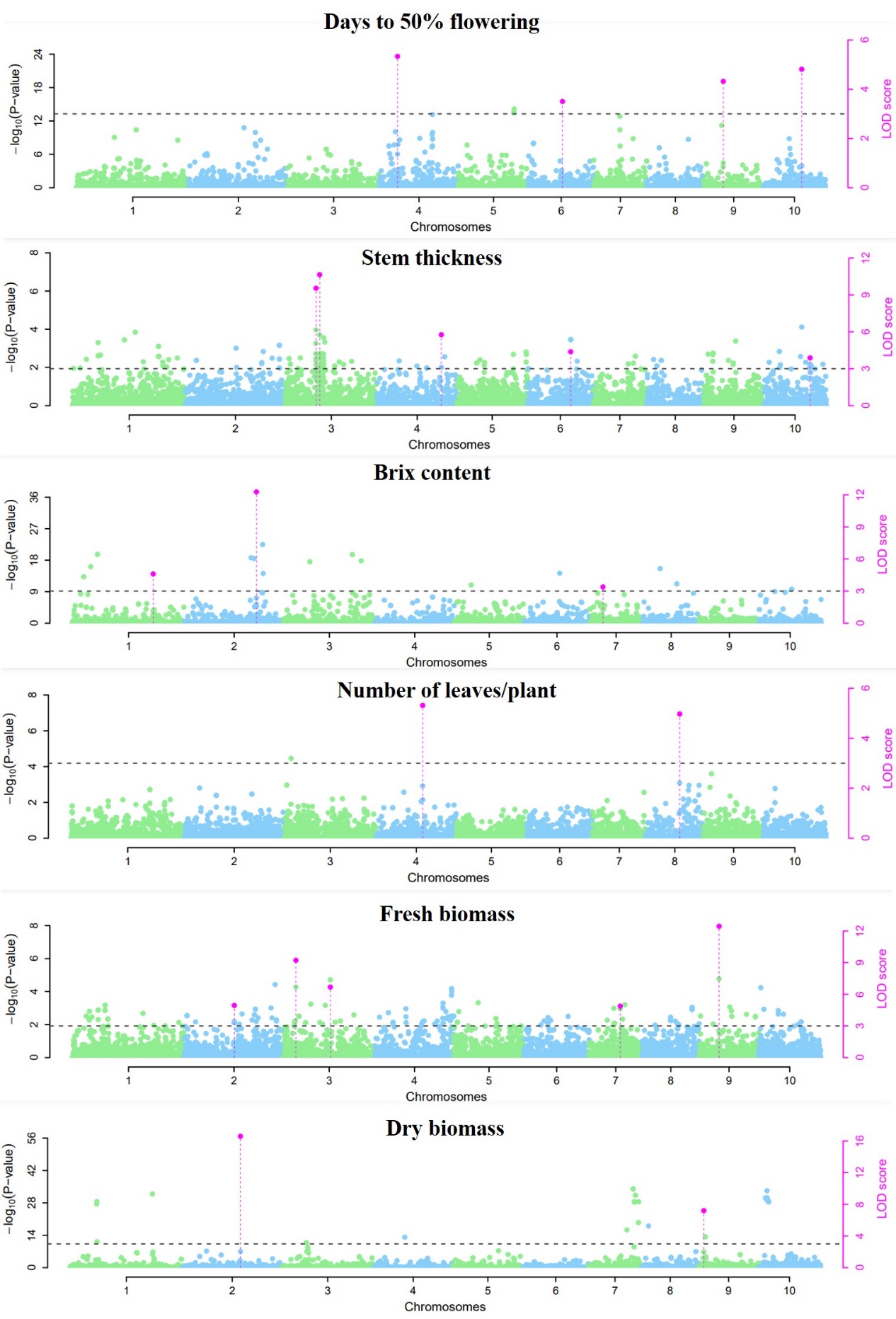
**

**Supplementary Figure 3.** Genome-wide association studies on six traits evaluated in this study using 3VmrMLM model. Pink dots represent QTNs having strong and independent effects on the respective traits.


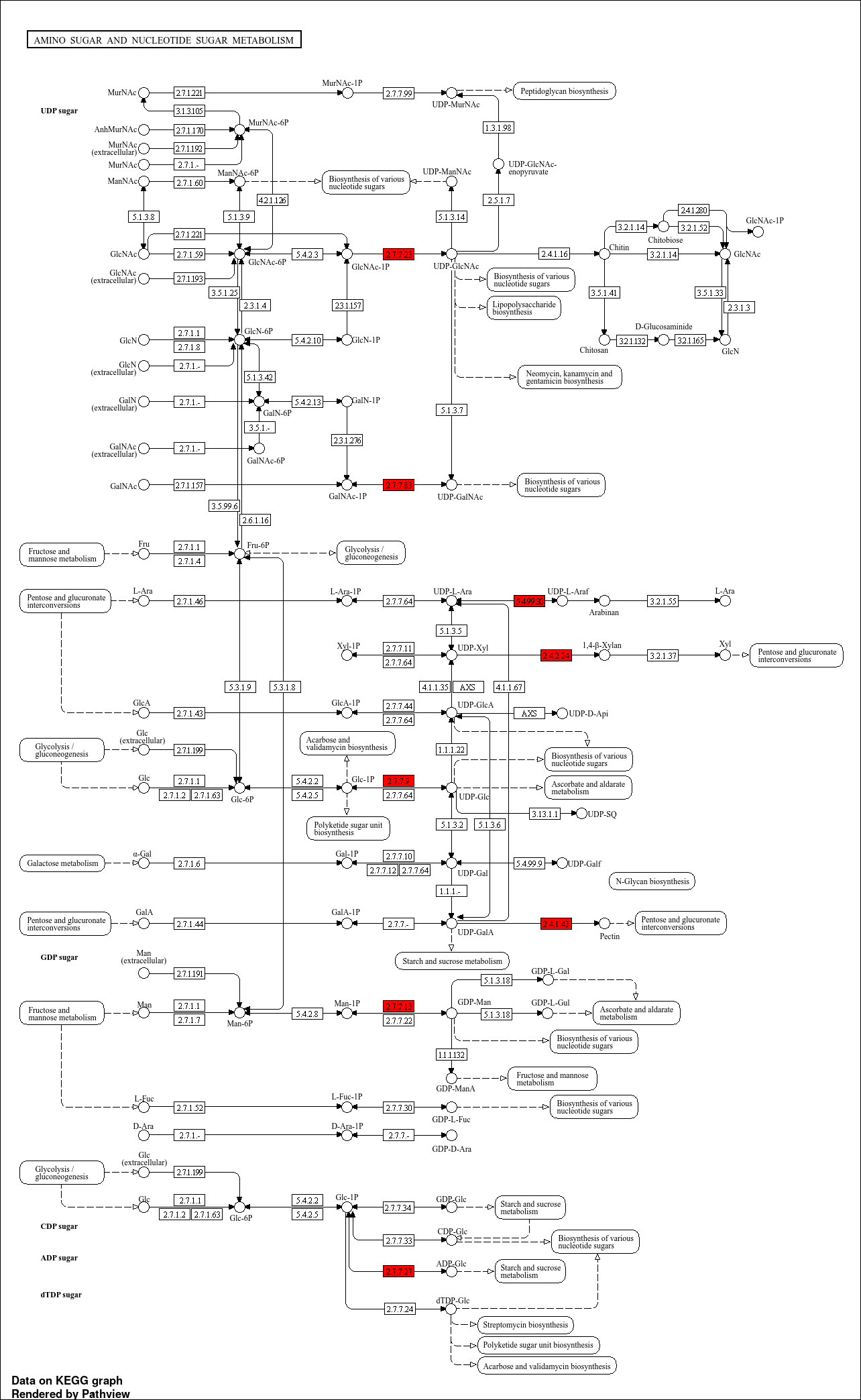


**Supplementary Figure 4.** Metabolic pathway of amino sugar and nucleotide sugar biosynthesis identified from this study GWAS highlighting (in red) candidate genes potential in the pathway

# Supplementary Tables

**Supplementary Table 1.** List of sweet sorghum accessions and the mean performance of the phenotypic traits used for GWAS in this study

| **Accessions** | **Origin** | **Name** | **DF** | **DM** | **PH** | **ST** | **NL** | **FB** | **DB** | **BX** |
| --- | --- | --- | --- | --- | --- | --- | --- | --- | --- | --- |
| PI144134 | South Africa | Inyangentombi | 92.67 | 171.5 | 211.5 | 11.5 | 13.17 | 706.17 | 297.17 | 8.83 |
| PI144331 | South Africa | ISIDOMBA | 104.17 | 177.5 | 212.83 | 14.17 | 12.5 | 713.67 | 328.67 | 4.5 |
| PI145619 | South Africa | ISIDOMBA | 83 | 167.67 | 262.17 | 9 | 11.83 | 957.17 | 348.83 | 11.83 |
| PI145622 | South Africa | Jiba | 121 | 184 | 264.83 | 6 | 14 | 712.67 | 324.83 | 12.67 |
| PI145632 | South Africa | TEGEVINI | 95.5 | 171.67 | 247.5 | 5.17 | 10.17 | 751.83 | 312.83 | 10.67 |
| PI145633 | South Africa | Tugela Ferry | 120.67 | 183 | 207.33 | 12 | 11.5 | 707.17 | 328.5 | 13.5 |
| PI146890 | Central Africa | SUGAR DRIP | 83 | 168 | 282.5 | 5 | 10.67 | 933.67 | 308.83 | 8.67 |
| PI147200 | South Asia | W. 21 | 85.67 | 168.17 | 263 | 21.5 | 11.67 | 704.67 | 309.5 | 7.17 |
| PI147224 | South Asia | B. 35 | 119.17 | 185 | 217.67 | 9.33 | 14.67 | 715 | 323.5 | 8.5 |
| PI147573 | Europe | MN 600 | 83.17 | 167.67 | 191.67 | 7.5 | 11.5 | 923.5 | 302.17 | 11.17 |
| PI149830 | East Africa | IS 2462 | 107.5 | 178.5 | 219.83 | 13.83 | 10 | 725.33 | 308.67 | 10.5 |
| PI149832 | East Africa | IS 2464 | 83.33 | 167.5 | 252.67 | 9 | 10.67 | 732.83 | 316.67 | 9.17 |
| PI152596 | North Africa | ANKOLIB TEQUIL | 85 | 167.5 | 258.5 | 10.83 | 10.33 | 717.83 | 314.33 | 10.33 |
| PI152629 | North Africa | Feterita Fayoumi D.S. 8 | 111 | 180.17 | 250.5 | 10.67 | 9.5 | 721.5 | 321.67 | 9.67 |
| PI152630 | North Africa | FETERITA FAYOUMI D.S. 10 | 113.5 | 180 | 210.83 | 5.83 | 13.5 | 943.33 | 305.67 | 6.83 |
| PI152633 | North Africa | FETERITA FAYOUMI D.S. 13 | 85.5 | 167.5 | 241.33 | 7.17 | 10 | 785.5 | 321.83 | 5.83 |
| PI152646 | North Africa | FETERITA GEZIRA | 122 | 185.5 | 192.83 | 20 | 13.33 | 738.33 | 330.5 | 5.67 |
| PI152650 | North Africa | FETERITA FULLI | 83.67 | 167.67 | 225.33 | 6.83 | 11 | 831 | 341.5 | 11 |
| PI152651 | North Africa | Feterita Geshaish | 88 | 169 | 223 | 6.83 | 8.83 | 705.83 | 301.5 | 12.17 |
| PI152671 | North Africa | GISHISH | 108.5 | 177.5 | 242.33 | 10.33 | 10 | 680 | 356.17 | 10 |
| PI152675 | North Africa | Heger Taie | 120 | 185 | 203.17 | 6.67 | 9.5 | 782.5 | 343.33 | 8.83 |
| PI152676 | North Africa | HEGIRI 1 | 90.5 | 169.5 | 195.83 | 21 | 9.5 | 873.5 | 324.5 | 9.17 |
| PI152683 | North Africa | HEMAISI RED SHENDI SHERSHER | 120 | 184.5 | 271.33 | 9 | 8.83 | 707.5 | 320.67 | 8 |
| PI152692 | United States | KAFIR PINK | 112.83 | 180.67 | 213 | 9.17 | 12.83 | 774 | 318.67 | 9.83 |
| PI152714 | North Africa | LUEL | 83 | 168.5 | 241.83 | 10.83 | 12.33 | 756.17 | 362.33 | 11.33 |
| PI152725 | North Africa | MALWAL AWEIL | 117.5 | 182.17 | 251.83 | 11.83 | 16 | 995.83 | 348.5 | 10.33 |
| PI152733 | North Africa | MERISSA (BARI) | 83.33 | 167.5 | 261.17 | 6.67 | 10 | 765.83 | 345.5 | 12.83 |
| PI152755 | North Africa | POTCH 4 | 95.5 | 172.5 | 185.67 | 8.67 | 13.33 | 928.33 | 338.67 | 10.17 |
| PI152764 | North Africa | QUERY 3 | 91 | 172 | 229.33 | 7.83 | 11 | 799.17 | 310.83 | 8.17 |
| PI152771 | North Africa | RAHMETALLA GALLABAT | 93.17 | 172.67 | 199.83 | 14.5 | 10.33 | 750.5 | 310.67 | 6.17 |
| PI152813 | North Africa | Wad Aker Red | 106.83 | 177.33 | 253.67 | 21.17 | 10.67 | 896 | 356.5 | 8.17 |
| PI152816 | North Africa | WAD FUR WHITE | 111 | 177.5 | 204 | 10.5 | 8.5 | 836.17 | 328.17 | 11.33 |
| PI152828 | Central Africa | U.T. 23 | 86 | 168.5 | 222.5 | 11.67 | 9.17 | 772.5 | 323.17 | 10.33 |
| PI152860 | North Africa | MERASI | 83 | 167.5 | 171.67 | 8.33 | 13.67 | 782.17 | 325.17 | 7.83 |
| PI152872 | North Africa | FETERITA ABDEL MAGID | 107.83 | 179 | 225.67 | 7 | 13 | 831.83 | 314.33 | 9 |
| PI152880 | North Africa | LWEL FADIANG | 87.17 | 168.67 | 170.83 | 10 | 11.67 | 888.33 | 355.5 | 9.5 |
| PI152909 | East Africa | Mahananga | 85 | 169 | 223.83 | 10.33 | 10.33 | 803 | 328.17 | 10 |
| PI152914 | United States | WAXY CLUB | 115 | 182 | 222.83 | 13 | 10.83 | 812 | 341.83 | 13.67 |
| PI152923 | North Africa | Duro El Jack | 93.5 | 171.83 | 237 | 11.67 | 8.5 | 771 | 332.33 | 12.5 |
| PI152953 | North Africa | CHIKKORI | 99 | 173 | 216.83 | 10.33 | 15.83 | 815.17 | 312.17 | 10.67 |
| PI152961 | North Africa | MALNAL | 85.5 | 169 | 227.17 | 9 | 10.83 | 803.5 | 348 | 12.17 |
| PI152963 | North Africa | Thok (B) | 119 | 184.83 | 256.5 | 17.67 | 11 | 952.83 | 329.83 | 11.67 |
| PI152966 | North Africa | Ayuak | 92.67 | 171.5 | 279.83 | 7 | 12.83 | 913.67 | 311 | 10.67 |
| PI152998 | East Africa | GUMBILU | 83.33 | 168 | 176 | 9.33 | 12.33 | 923.17 | 346.83 | 8.83 |
| PI153871 | East Africa | MUBEYA | 106.5 | 181 | 223.17 | 10.33 | 11.5 | 964.17 | 330 | 9.33 |
| PI154750 | East Africa | Serere | 91 | 171 | 190.67 | 9.17 | 12.17 | 980.33 | 349.5 | 10.83 |
| PI154787 | East Africa | MN 1344 | 121.33 | 186 | 209.5 | 20.67 | 7.83 | 925.67 | 286.5 | 12 |
| PI154796 | East Africa | NKUMBA | 83 | 168.17 | 239.17 | 9 | 8.5 | 885 | 345.67 | 13.17 |
| PI154800 | East Africa | Wenabu | 117.5 | 186 | 229.67 | 9.33 | 9.17 | 909.17 | 315 | 9 |
| PI154844 | East Africa | 'GRASSL' | 119.67 | 186 | 293.17 | 9 | 11.17 | 906.83 | 318.17 | 6.83 |
| PI154846 | East Africa | KABIRI | 86.17 | 168.33 | 247.5 | 10.83 | 13 | 924 | 315.83 | 9 |
| PI154929 | East Africa | J56 Akouangok | 121 | 185.67 | 237.17 | 10 | 11.33 | 956.17 | 313 | 10 |
| PI154943 | East Africa | L28 Lawere | 83 | 169 | 220.67 | 12 | 9.67 | 917.33 | 328.5 | 11 |
| PI154944 | East Africa | L31 Emiroit | 118 | 184.5 | 253.67 | 12.5 | 10.33 | 979.67 | 298.33 | 12.33 |
| PI154962 | East Africa | V3 Nakyeru | 119 | 183.83 | 270.5 | 20.67 | 9.17 | 956.5 | 313.17 | 10.33 |
| PI154980 | East Africa | Wheatland | 90.67 | 172 | 192.17 | 8 | 11.17 | 967.83 | 362.83 | 11.67 |
| PI154987 | South Africa | S. A. 1 | 120.67 | 184.5 | 231.5 | 8.33 | 10.33 | 866.83 | 321.17 | 7.5 |
| PI154988 | South Africa | S. A. 2 | 109.67 | 178.5 | 205.5 | 10.83 | 10.67 | 856.5 | 322.83 | 9.33 |
| PI154990 | South Africa | P 127 (S.A. 5) | 83 | 167.5 | 315 | 8.83 | 12.67 | 890 | 310.67 | 8.67 |
| PI155336 | East Africa | MUYO | 122 | 186 | 237.67 | 11.33 | 10.83 | 927.33 | 321.33 | 11 |
| PI155485 | South Africa | Maila | 92.5 | 170 | 243.17 | 11 | 12 | 900.83 | 327 | 14.5 |
| PI155516 | South Africa | MASAKA | 120 | 185.67 | 230.67 | 19.17 | 8.5 | 835 | 322.5 | 10.67 |
| PI155543 | South Africa | Hasesa | 120 | 185 | 208.5 | 9.83 | 8.17 | 891.33 | 315.17 | 14.5 |
| PI155556 | South Africa | MAILA | 83 | 167.5 | 190.33 | 11 | 11 | 769 | 311.67 | 10.67 |
| PI155571 | South Africa | LONGWE | 120 | 186 | 246.33 | 20.67 | 11.5 | 939.5 | 330 | 9.5 |
| PI155609 | South Africa | MAPIERA | 111 | 180 | 272 | 11 | 13.33 | 845.17 | 322.67 | 9.5 |
| PI155760 | East Africa | Namuse | 119 | 184 | 275.67 | 15.5 | 10.5 | 947.17 | 363.5 | 6.83 |
| PI155805 | East Africa | MAPIRA | 96.83 | 172.83 | 181 | 10.83 | 10.67 | 937.5 | 332.67 | 5 |
| PI155845 | East Africa | MN 2077 | 120 | 184.33 | 240.17 | 9.17 | 12.5 | 858.83 | 337 | 13.67 |
| PI155902 | East Africa | MN 2103 | 83.67 | 168 | 228.33 | 13.17 | 10.67 | 907 | 346.5 | 12.17 |
| PI155924 | South Africa | CHIFUNGO | 113.5 | 179.5 | 226 | 21.5 | 9.67 | 893.33 | 308.83 | 15.5 |
| PI156136 | South Africa | MAILA | 120 | 186 | 193.5 | 7.67 | 9.67 | 975.67 | 345.67 | 12.33 |
| PI156203 | East Africa | MN 2089 | 83.5 | 167.67 | 218.5 | 16.17 | 10.5 | 903.83 | 349.67 | 5.5 |
| PI156217 | East Africa | MN 2109 | 120 | 186 | 241.83 | 9 | 12.67 | 870 | 316.33 | 7 |
| PI156252 | East Africa | Nefee | 117.5 | 183.33 | 203.33 | 11 | 8.67 | 990.5 | 341.17 | 10.5 |
| PI156352 | South Africa | MN 2238 | 83.33 | 168 | 270.17 | 11.33 | 10.33 | 919.83 | 325.5 | 12.17 |
| PI156356 | South Africa | Sonkwe | 120 | 185.83 | 255.17 | 9.5 | 11.5 | 890.17 | 330 | 12.33 |
| PI156393 | East Africa | MN 2277 | 87 | 168.5 | 215.17 | 21.5 | 10.83 | 897.67 | 319.17 | 11.5 |
| PI157030 | East Africa | Andiwo III 57 | 83 | 168.33 | 239.5 | 8 | 11.33 | 867.5 | 314.83 | 10.67 |
| PI157033 | East Africa | Ifube No. 18 | 113.5 | 182.5 | 216.17 | 10.33 | 11.17 | 914.17 | 338.33 | 13.67 |
| PI157035 | East Africa | Nyagwang No. 56 | 122 | 177 | 217.5 | 11 | 9.83 | 873.17 | 333.17 | 9.17 |
| PI157804 | North Africa | Feterita Abu Derega | 87.5 | 168.67 | 192.17 | 9.5 | 10.5 | 856.5 | 300.83 | 10.33 |
| PI167047 | Middle East | AKDARI | 101 | 176 | 316.83 | 14.5 | 9.33 | 912.33 | 305.17 | 10.33 |
| PI167352 | Middle East | AKDARI | 83.17 | 167.5 | 298.67 | 12 | 14 | 856.83 | 317.5 | 9.17 |
| PI170783 | Middle East | AKDARI | 122 | 186 | 228.33 | 9.67 | 10 | 958.17 | 289.17 | 13.67 |
| PI170787 | Middle East | MN 2826 | 106.5 | 178.5 | 271.17 | 8 | 12.5 | 841 | 288.33 | 14 |
| PI170802 | Middle East | IS 12807 | 120 | 185 | 275.5 | 9.67 | 10.5 | 934.83 | 342.17 | 12.5 |
| PI170805 | Middle East | IS 12810 | 83.33 | 168 | 212 | 10 | 8.5 | 852.17 | 326.83 | 8 |
| PI173112 | Middle East | 7392 | 122 | 186 | 243.17 | 9 | 6.83 | 892.5 | 282.33 | 7.5 |
| PI173118 | Middle East | 8371 | 105 | 172.33 | 272.83 | 11 | 8.83 | 876.5 | 296.67 | 15 |
| PI173120 | Middle East | 8493 | 122 | 185.83 | 306.83 | 13.83 | 11.5 | 899.17 | 306 | 7.5 |
| PI173808 | Middle East | GILGIL | 101 | 176 | 264.67 | 9.67 | 9.67 | 893.83 | 354.5 | 12.67 |
| PI173971 | South Asia | JAWAR | 110 | 178.5 | 184.5 | 21.17 | 10.83 | 943.5 | 322.5 | 10.67 |
| PI174381 | Middle East | KARADARI | 120.67 | 186 | 228.5 | 7.83 | 8.33 | 908.17 | 313.17 | 8.5 |
| PI17548 | Australia | 'RED AMBER' | 92.5 | 169.33 | 174.67 | 10.33 | 9.5 | 717.17 | 316.5 | 8.17 |
| PI175919 | Middle East | IS 12833 | 83 | 167.5 | 201.17 | 8.17 | 11.33 | 910.17 | 326.33 | 7.5 |
| PI177156 | Middle East | MN 2742 | 119.67 | 186 | 236.17 | 7.33 | 10.67 | 924.17 | 338.33 | 14.83 |
| PI177553 | Middle East | AKDARI | 83 | 167.5 | 205.83 | 16.5 | 8.83 | 928.67 | 339.67 | 5.5 |
| PI177554 | Middle East | IS 12856 | 122 | 185.33 | 235.83 | 11.5 | 10.17 | 958.83 | 303.17 | 7.5 |
| PI179504 | Middle East | AKDARI | 92 | 170 | 240.5 | 10.33 | 9.17 | 938 | 313.83 | 9 |
| PI179747 | South Asia | JAWAR | 122 | 186 | 262.83 | 9.33 | 9.33 | 933.83 | 344.5 | 9 |
| PI179749 | South Asia | Juar | 83 | 167.83 | 244.83 | 8.17 | 12.33 | 843.33 | 290.83 | 9.17 |
| PI180004 | South Asia | JAWAR | 102.17 | 177.5 | 213.5 | 6.67 | 11 | 939 | 351.67 | 10.17 |
| PI180005 | South Asia | JAWAR | 84.5 | 167.5 | 200.67 | 20.33 | 10.5 | 850.17 | 374.83 | 5.5 |
| PI180348 | South Asia | Juar | 105.5 | 177.5 | 277.33 | 9 | 10.83 | 917 | 328.67 | 11.5 |
| PI180489 | South Asia | Juar | 83 | 167.5 | 215 | 10.83 | 11.17 | 870.17 | 320.83 | 14 |
| PI181077 | South Asia | DEPAR | 121 | 186 | 237.33 | 15 | 10.17 | 930 | 320.83 | 12.17 |
| PI181080 | South Asia | HONEY SORGHUM | 90 | 170.17 | 206.33 | 10 | 10 | 914 | 375.83 | 12 |
| PI181083 | South Asia | KAMANDRI | 119.67 | 184.33 | 199.67 | 10.83 | 10.33 | 969.83 | 339.5 | 9 |
| PI181899 | Middle East | IS 12893 | 83 | 167.5 | 250 | 9 | 10.83 | 929.83 | 405.17 | 7 |
| PI182303 | Middle East | AKDARI | 103.5 | 177.17 | 258.67 | 12.67 | 9.33 | 883.5 | 329.17 | 8.5 |
| PI183149 | South Asia | JUAR | 98 | 174.5 | 226.67 | 15 | 12 | 946.83 | 341.33 | 10.17 |
| PI189114 | Central Africa | MN 2972 | 120 | 186 | 278.5 | 10.67 | 9.67 | 941.5 | 370.83 | 10 |
| PI195754 | East Asia | KAOLIANG | 83.33 | 168.5 | 285.67 | 10.5 | 11.83 | 931 | 328.67 | 7.17 |
| PI196049 | East Africa | IS 2131 | 120 | 185.67 | 229.33 | 9 | 10.5 | 949.33 | 312 | 5.5 |
| PI196592 | East Asia | MN 3089 | 120.67 | 184 | 255 | 11.17 | 9.67 | 918 | 304.33 | 7.33 |
| PI196598 | East Asia | MN 3095 | 91 | 171 | 246.83 | 11 | 11.83 | 945.17 | 367 | 9.33 |
| PI197542 | North Africa | SUCRE DROME | 120.5 | 184.5 | 222.33 | 11.83 | 10.33 | 954.67 | 340.67 | 10.67 |
| PI198885 | Australia | Sweet Saccaline | 105 | 178.5 | 256.67 | 13.5 | 10 | 962.33 | 347.33 | 10.17 |
| PI201723 | Central Africa | FETERITA LA ESTENZUELA | 83 | 167.5 | 209.33 | 14.67 | 11.33 | 967.33 | 363.83 | 11 |
| PI217691 | North Africa | NAGAD EL MUR | 101 | 175.5 | 223.5 | 9 | 10.33 | 941.33 | 339.5 | 5.5 |
| PI217770 | North Africa | BARGOWI | 120 | 185.67 | 290.83 | 21.17 | 12 | 988.83 | 371.83 | 7.33 |
| PI218112 | South Asia | IS 2352 | 85.83 | 168.67 | 258.17 | 9.17 | 9.17 | 961.33 | 308 | 8 |
| PI221560 | Central Africa | BALAKA | 120 | 186 | 229.5 | 10 | 8 | 931.83 | 314.67 | 8.5 |
| PI247136 | Europe | MN 4052 | 120 | 186 | 257.67 | 10.67 | 12.17 | 941.5 | 370.83 | 10 |
| PI247745 | Central Africa | Tjolotjo | 83.33 | 168.5 | 253.83 | 10.5 | 12.17 | 931 | 328.67 | 7.17 |
| PI248298 | South Asia | 'CHINESE AMBER' | 120 | 185 | 226 | 11 | 9.5 | 928.33 | 353.5 | 8 |
| PI250234 | South Asia | MN 4120 | 120 | 186 | 236.5 | 13 | 10.67 | 988.67 | 420 | 12.33 |
| PI250402 | South Asia | IS 13122 | 115 | 180.67 | 269 | 14.33 | 12.5 | 941.5 | 306.67 | 11 |
| PI250521 | South Asia | MN 4122 | 83 | 167.5 | 280.83 | 10.17 | 8.67 | 922 | 330.33 | 14.5 |
| PI250582 | North Africa | MN 4124 | 102.67 | 177 | 293.83 | 9.5 | 12.67 | 954.83 | 317.5 | 10.33 |
| PI250897 | Middle East | MN 4133 | 122 | 185.67 | 241.33 | 10.33 | 11.5 | 940.33 | 322.17 | 8.5 |
| PI250898 | Middle East | MN 4134 | 86 | 169 | 227.33 | 9.17 | 8.5 | 950.67 | 308.5 | 10.5 |
| PI251672 | Europe | MN 4135 | 122 | 184.83 | 247.83 | 10 | 9.67 | 932.33 | 368.17 | 10.17 |
| PI253795 | Middle East | IS 13148 | 111.33 | 177 | 274.83 | 19.67 | 12 | 968 | 339 | 11 |
| PI253796 | Middle East | IS 13149 | 83 | 168 | 218.67 | 13.17 | 10 | 913.5 | 354.83 | 10.67 |
| PI253986 | Middle East | IS 13150 | 122 | 186 | 199.67 | 9.67 | 9.5 | 950 | 309.83 | 8.83 |
| PI255239 | Others | CAXA | 90.5 | 170.67 | 202.5 | 9.83 | 11 | 941.83 | 344.33 | 5.5 |
| PI257599 | East Africa | NO. 5 GAMBELA | 115 | 181 | 224.33 | 15.5 | 11.83 | 905.5 | 305.67 | 5 |
| PI257600 | East Africa | NO. 6 GAMBELA | 83 | 168 | 272.83 | 9.33 | 10.33 | 955.83 | 334.83 | 10.17 |
| PI257602 | East Africa | NO. 8 GAMBELA | 121.33 | 185 | 274 | 11.17 | 11.67 | 952.33 | 298.83 | 11.33 |
| PI260210 | Europe | Darso 28 | 92.5 | 171.5 | 211 | 12.67 | 11 | 948.33 | 301.5 | 8.5 |
| PI273955 | East Africa | MN 4566 | 115 | 180 | 300 | 10.67 | 10.33 | 951.5 | 309.67 | 6.5 |
| PI287625 | South Africa | IS 12295 | 120 | 186 | 223.5 | 9.5 | 12.17 | 973.5 | 332.83 | 10.83 |
| PI287627 | South Africa | IS 13478 | 90.33 | 169.67 | 306.67 | 10.83 | 11.33 | 934.33 | 307.5 | 7.33 |
| PI302120 | Europe | IS 13716 | 101 | 174.83 | 260.17 | 20.83 | 13.17 | 946.17 | 338.17 | 12.83 |
| PI302131 | Europe | MN 3998 | 83 | 167.5 | 205.17 | 12.67 | 11.33 | 945 | 324.33 | 15 |
| PI302198 | Others | IS 14152 | 120.67 | 186 | 236.5 | 15 | 9.83 | 914.33 | 348.17 | 16 |
| PI302199 | Others | IS 14153 | 95 | 172.17 | 275.5 | 11 | 11.67 | 950.17 | 321.17 | 11.83 |
| PI302252 | East Asia | IS 13726 | 122 | 186 | 233.67 | 20.33 | 15 | 908.5 | 342.17 | 8.17 |
| PI302264 | East Africa | MN 4330 | 92.5 | 170.5 | 213 | 8.33 | 14.5 | 962.17 | 331.67 | 5.5 |
| PI303658 | North Africa | Nerum Boer | 107.5 | 175.83 | 197.17 | 15.5 | 10.5 | 943 | 334.33 | 14.67 |
| PI48191 | Australia | SACCALINE | 87.17 | 168.5 | 213.5 | 9.17 | 12.83 | 902.83 | 363.83 | 8 |
| PI511355 | United States | 'SMITH' | 90 | 169.67 | 270.5 | 15 | 10.83 | 960.67 | 337.33 | 5 |
| PI52606 | South Africa | IS 12724 | 122 | 185 | 162.5 | 9 | 10.17 | 739.5 | 334 | 9.83 |
| PI533998 | United States | Brawley | 83.33 | 168.5 | 139.33 | 11 | 11.17 | 947.17 | 337.17 | 7.67 |
| PI535783 | United States | N98 | 122 | 186 | 205 | 10.33 | 10.83 | 907.67 | 330.33 | 6.67 |
| PI535785 | United States | N100 | 91.17 | 171 | 239.67 | 18 | 9.17 | 974.5 | 325.33 | 6.5 |
| PI535792 | United States | N107 | 120.33 | 185 | 246.67 | 9.17 | 12.17 | 903.5 | 341.83 | 10.33 |
| PI535796 | United States | N111 | 83 | 167.5 | 222 | 9.33 | 10.33 | 970.83 | 359.5 | 13.67 |
| PI562716 | United States | HONEY NO. 2' | 118 | 184.67 | 244.17 | 7.33 | 8.67 | 944.17 | 348.17 | 10.5 |
| PI563295 | United States | 'RIO' | 91.67 | 171.17 | 217.83 | 10.5 | 12.83 | 942.17 | 362.83 | 11 |
| PI566819 | United States | 'DELLA' | 120 | 186 | 288 | 10 | 11.33 | 954.17 | 323.83 | 8.67 |
| PI583832 | United States | 'TOP 76-6' | 120 | 184.5 | 265.33 | 6.33 | 9.67 | 982.67 | 324.17 | 10 |
| PI584989 | United States | 'POPSORGHUM' | 83 | 167.5 | 284.5 | 11 | 9 | 972.17 | 332.5 | 10.67 |
| PI586443 | Europe | IS 27818 | 117.67 | 183.67 | 241.33 | 15.17 | 11.5 | 952.83 | 332.17 | 12 |
| PI586541 | Australia | TRACY' | 95.5 | 172.5 | 216.5 | 9.5 | 11.67 | 938.67 | 351.67 | 13.5 |
| PI610727 | East Asia | GAI GAOLIANG | 102.5 | 175.17 | 226.83 | 11.33 | 10 | 981 | 325.33 | 12.17 |
| PI641806 | United States | AMES AMBER | 101 | 174.5 | 210.83 | 10.33 | 11.17 | 934.33 | 318.83 | 14.17 |
| PI641807 | United States | ATLAS | 122 | 185.67 | 241.5 | 8.33 | 8.67 | 926.83 | 271.33 | 14.83 |
| PI641815 | United States | EARLY FOLGER | 83.33 | 168.67 | 213.17 | 11 | 12.33 | 971.5 | 315.33 | 8.17 |
| PI641817 | United States | 'EARLY SUMAC' | 120 | 184.5 | 305 | 15 | 12.83 | 926.83 | 367.17 | 9.17 |
| PI641821 | United States | HONEY DRIP | 93.5 | 172 | 244.5 | 15.17 | 10.33 | 968.83 | 326.83 | 7.17 |
| PI641834 | United States | 'PLANTER' | 103.83 | 176 | 211.33 | 9.17 | 13.17 | 945.17 | 314.5 | 7.5 |
| PI641835 | United States | REX' | 120.33 | 186 | 230.5 | 10.67 | 11.5 | 953 | 372.33 | 8.83 |
| PI641848 | United States | TEXAS SEEDED RIBBON | 83 | 167.5 | 182.5 | 8 | 9.17 | 981.83 | 333.67 | 7.33 |
| PI641862 | United States | COLLIER | 113.5 | 179.83 | 202.5 | 10.33 | 9.67 | 952.17 | 335 | 6 |
| PI641893 | United States | DWARF ASHBURN | 89 | 169 | 262.17 | 11.5 | 9.67 | 950.83 | 380.5 | 5 |
| PI641904 | United States | H.C. 41-13 | 120.17 | 186 | 210.67 | 9.5 | 8 | 937 | 341.5 | 7.17 |
| PI641909 | North Africa | Red Losinga | 91.17 | 170.67 | 259.83 | 10 | 11.33 | 984.17 | 310.33 | 11.67 |
| PI88000 | East Asia | KAOLIANG-WX | 83 | 167.5 | 186.5 | 7.67 | 9.33 | 619.5 | 313.33 | 5.5 |
| PI88007 | East Asia | Bangu manguisusu | 121.33 | 186 | 218.33 | 11 | 11.33 | 708.17 | 313.83 | 9.17 |
| PI92270 | East Asia | IS 12748 | 85.5 | 167.83 | 188.5 | 8.17 | 12.67 | 960.5 | 340.33 | 8.83 |

DF- Days to 50% flowering, DM- Days to maturity, PH- plant height (cm), ST- Stem thickness (mm), NL- Number of leaves/plant, FB- Fresh biomass (g), DB- Dry biomass (g), BX- Brix content (%).

**Supplementary Table 2.** Phenotypic diversity observed in the sweet sorghum panel

| **Traits** | **Mean±SE** | **Range** | **SD** | **CV %** | **Heritability** |
| --- | --- | --- | --- | --- | --- |
| Days to flowering | 102.84±1.14 | 83 ­- 122 | 15.47 | 15.05 | 0.94 |
| Days to maturity | 176.51±0.54 | 167.5 -­ 186 | 7.36 | 4.17 | 0.43 |
| Plant height (cm ) | 236.86±2.13 | 139.33 - ­ 316.83 | 28.85 | 12.18 | 0.4 |
| Stem thickness (mm) | 11.34±0.28 | 5 ­- 21.5 | 3.729 | 32.88 | 0.93 |
| Number of leaves/ plant | 10.86±0.12 | 6.83 -­ 16 | 1.575 | 14.5 | 0.45 |
| Fresh biomass | 892.87±6.03 | 619.5 -­ 995.83 | 81.62 | 9.14 | 0.7 |
| Dry biomass | 329.66±1.63 | 271.33 -­ 420 | 22.04 | 6.69 | 0.72 |
| Brix content (%) | 9.83±0.19 | 4.5 ­- 16 | 2.539 | 25.83 | 0.83 |

SE- Standard error, SD- Standard deviation, CV- Coefficient of variance.

**Supplementary Table 3.** Comprehensive list of candidate genes identified in proximity to the significant quantitative trait nucleotides (QTNs).

| **Trait** | **QTN** | **Candidate gene** | **Annotation** |
| --- | --- | --- | --- |
| Days to flowering | S04_9611322 | *Sobic.004G102700* | BES1/BZR1 homolog protein, putative, expressed |
|  |  | *Sobic.004G102400* | similar to LAs17 Binding protein |
|  |  | *Sobic.004G102500* | BES1/BZR1 plant transcription factor |
|  |  | *Sobic.004G103000* | U-BOX DOMAIN-CONTAINING PROTEIN 17 |
|  |  | *Sobic.004G103400* | Leucine Rich Repeat |
|  | S06_49330184 | *Sobic.006G128200* | Threonine-specific protein kinase |
|  |  | *Sobic.006G126100* | SERINE/THREONINE PROTEIN PHOSPHATASE |
|  |  | *Sobic.006G126300* | GLUCOSE-6-PHOSPHATE 1-DEHYDROGENASE |
|  |  | *Sobic.006G126600* | ZINC FINGER PROTEIN |
|  | S09_10767169 | *Sobic.009G080000* | SQUAMOSA PROMOTER-BINDING-LIKE PROTEIN 14-RELATED |
|  | S10_47190193 | *NA* | NA |
| Stem thickness | S03_14723140 | *Sobic.003G144400* | Homeodomain-like |
|  | S03_23932008 | *Sobic.003G165800* | UDP-N-acetylglucosamine--N-acetylmuramyl-pyrophosphor yl-undecaprenol N-acetylglucosamine transferase, putative, expressed |
|  | S04_62493744 | *Sobic.004G283201* | AP2 domain |
|  |  | *Sobic.004G282900* | Predicted transporter/transmembrane protein |
|  |  | *Sobic.004G283300* | ETHYLENE-RESPONSIVE TRANSCRIPTION FACTOR |
|  |  | *Sobic.004G283400* | RING FINGER DOMAIN-CONTAINING |
|  | S06_52891675 | *Sobic.006G173000* | transporter, major facilitator family |
|  | S10_53386896 | *Sobic.010G192200* | two-component response regulator ARR-B family |
|  |  | *Sobic.010G191900* | two-component response regulator ARR-B family |
| Brix content | S01_67476108 | *Sobic.001G387600* | ethylene-insensitive 3 |
|  |  | *Sobic.001G387000* | RING ZINC FINGER PROTEIN |
|  |  | *Sobic.001G387300* | EXOSTOSIN HEPARAN SULFATE GLYCOSYLTRANSFERASE |
|  | S02_67296984 | *Sobic.002G295800* | Protein kinase domain |
|  |  | *Sobic.002G295200* | RBR FAMILY RING FINGER AND IBR DOMAIN-CONTAINING |
|  |  | *Sobic.002G295700* | Synaptic vesicle transporter SV2 |
|  | S07_5383507 | *Sobic.007G053100* | amino acid permease family protein |
|  |  | *Sobic.007G053500* | MOB kinase activator 1 |
|  |  | *Sobic.007G052900* | Histone acetyltransferase |
|  |  | *Sobic.007G052400* | MITOCHONDRIAL OUTER MEMBRANE PROTEIN 25 |
| Number of leaves/ plant | S04_56429277 | *Sobic.004G214500* | DNA repair protein RAD18 |
|  |  | *Sobic.004G214800* | Leucine Rich Repeat (LRR_1) |
|  |  | *Sobic.004G214100* | UDP-N-acetylglucosamine transporter |
|  | S08_53828669 | *NA* | NA |
| Fresh biomass | S02_58995294 | *Sobic.002G200100* | Zinc finger, C2HC5-type |
|  |  | *Sobic.002G200000* | MEMBRANE ASSOCIATED RING FINGER |
|  |  | *Sobic.002G200300* | No apical meristem (NAM) protein (NAM) |
|  | S03_5989832 | *Sobic.003G069950* | Protein kinase domain |
|  |  | *Sobic.003G069800* | NUCLEOPORIN-RELATED |
|  | S03_54843288 | *Sobic.003G214400* | Amino acid transporter |
|  |  | *Sobic.003G214000* | DNAJ HOMOLOG SUBFAMILY C MEMBER |
|  |  | *Sobic.003G214500* | SMALL UBIQUITIN-RELATED MODIFIER |
|  | S07_53586588 | *Sobic.007G125400* | Chitinase |
|  | S09_11063229 | *Sobic.009G080400* | GLUCOSYL/GLUCURONOSYL TRANSFERASES |
| Dry biomass | S02_61546596 | *Sobic.002G224100* | phosphate carrier protein, mitochondrial precursor |
|  |  | *Sobic.002G223700* | RHOMBOID-RELATED |
|  |  | *Sobic.002G223800* | similar to Hydroxyproline-rich glycoprotein-like protein |
|  |  | *Sobic.002G224000* | carbonic anhydras |
|  | S09_1695932 | *Sobic.009G018450* | Threonine-specific protein kinase |
|  |  | *Sobic.009G019100* | chitin elicitor-binding protein |

**Supplementary Table 4.** Haplotype diversity and allelic combination for candidate genes showing significant phenotype difference in different haplotype groups.

| **Candidate gene** | **SNPs present** | **Haplotype groups** | | | |
| --- | --- | --- | --- | --- | --- |
|  |  | **Hap1** | **Hap2** | **Hap3** | **Hap4** |
| Sobic.006G128200 | 2 SNPs | CC (52) | CT (81) | TC (50) | NA |
| Sobic.001G387600 | 7 SNPs | AGGATAA (12) | AGGATTG (21) | GAAGCAA (105) | GAAGCTG (45) |
| Sobic.003G069950 | 4 SNPs | AACT (50) | AATT (100) | GATT (33) | NA |
| Sobic.003G214400 | 3 SNPs | AAC (15) | AAG (15) | ACG (114) | CCG (39) |

Green color indicates the allelic combination of superior haplotype formed by the candidate genes. Number in parentheses indicate number of genotypes in each haplotype.

**Supplementary Table 5.** Haplotype analysis showing significant difference between the haplotypes for the candidate genes.

| **Trait** | **Candidate gene** | **Function** | **Mean value of different haplotypes** | | | | **p value** |
| --- | --- | --- | --- | --- | --- | --- | --- |
|  |  |  | **Hap1** | **Hap2** | **Hap3** | **Hap4** |  |
| Days to flowering | Sobic.006G128200 | Threonine-specific protein kinase | 102 (52) | 105 (81) | 98 (50) | NA | 0.04 |
| Brix content (%) | Sobic.001G387600 | ethylene-insensitive 3 | 14.72 (12) | 9.02 (21) | 9.56 (105) | 9.76 (45) | <0.01 |
| Fresh biomass (g) | Sobic.003G069950 | Protein kinase domain | 784.2 (50) | 929.15 (100) | 892.4 (33) | NA | <0.01 |
|  | Sobic.003G214400 | Amino acid transporter | 814.2 (15) | 931.6 (15) | 897.5 (114) | 889.2 (39) | <0.01 |

Green color indicates the allelic combination of superior haplotype formed by the candidate genes. Number in parentheses indicate number of genotypes in each haplotype.
